# Combined DNA Methylation and Transcriptomic Assessments to Determine a Prognostic Model for PD-1-Negative Hepatocellular Carcinoma

**DOI:** 10.3389/fcell.2021.708819

**Published:** 2021-08-11

**Authors:** Lixu Zhu, Wenzhi Guo

**Affiliations:** ^1^Department of Hepatobiliary and Pancreatic Surgery, The First Affiliated Hospital of Zhengzhou University, Zhengzhou, China; ^2^Key Laboratory of Hepatobiliary and Pancreatic Surgery and Digestive Organ Transplantation of Henan Province, The First Affiliated Hospital of Zhengzhou University, Zhengzhou, China; ^3^Open and Key Laboratory of Hepatobiliary & Pancreatic Surgery and Digestive Organ Transplantation at Henan Universities, Zhengzhou, China; ^4^Henan Key Laboratory of Digestive Organ Transplantation, Zhengzhou, China

**Keywords:** hepatocellular carcinoma, PD-1, methylation, ESTIMATE, prognosis

## Abstract

Hepatocellular carcinoma (HCC) has the highest incidence and mortality of any malignancy in the world. Immunotherapy has been a major breakthrough for HCC treatment, but immune checkpoint inhibitors (ICIs) are effective in only a small percentage of HCC patients. In the present study, we screened programmed cell death protein 1 (PD-1) -negative HCC samples, which are frequently resistant to ICIs, and identified their methylation and transcription characteristics through the assessment of differential gene methylation and gene expression. We also screened for potential targeted therapeutic drugs using the DrugBank database. Finally, we used a LASSO (least absolute shrinkage and selection operator) regression analysis to construct a prognostic model based on three differentially methylated and expressed genes (DMEGs). The results showed that ESTIMATE (Estimation of Stromal and Immune Cells in Malignant Tumors using Expression Data) scores for the tumor samples were significantly lower compared to normal sample ESTIMATE scores. In addition, we identified 31 DMEGs that were able to distinguish PD-1-negative samples from normal samples. A functional enrichment analysis showed that these genes were involved in a variety of tumor-related pathways and immune-related pathways, and the DrugBank screening identified potential therapeutic drugs. Finally, the prognostic model based on three DMEGs (*UBD*, *CD5L*, and *CD213A2*) demonstrated good predictive power for HCC prognosis and was verified using an independent cohort. The present study demonstrated the methylation characteristics of PD-1-negative HCC samples, identified several potential therapeutic drugs, and proposed a prognostic model based on *UBD*, *CD5L*, and *CD213A2* methylation expression. In conclusion, this work provides an in-depth understanding of methylation in HCC samples that are not sensitive to ICIs.

## Introduction

Hepatocellular carcinoma (HCC) represents the malignancy with the highest incidence and fatality rate in the world (especially in East Asia and Southern Africa), resulting in approximately 800,000 deaths per year (Forner et al., 2018). Although a variety of treatment methods (e.g., radiotherapy, chemotherapy, surgical treatment, and liver transplantation) have made great progress, the prognoses of patients with HCC have not improved significantly, mainly due to continued difficulty with early diagnosis, a high rate of recurrence, and limited indicators (El-Serag et al., 2008; Kawamura et al., 2020; Kudo, 2020b; Schoenberg et al., 2020). In recent years, immunotherapy has been a major breakthrough for HCC treatment (Li et al., 2019; Zhang Q. et al., 2020). With the development and application of immune checkpoint inhibitors (ICIs) targeting programmed cell death protein 1 (PD-1) and programmed death-ligand 1, the prognoses for some HCC patients have significantly improved (Kalathil et al., 2016; Mahn et al., 2020). However, immune escape and other related mechanisms within the tumor microenvironment have not been adequately studied, and only a small proportion of patients respond to immunotherapy (Sakuishi et al., 2010; Ng et al., 2020). Therefore, it is both urgent and necessary to further explore immune mechanisms related to HCC occurrence and development to identify new targets for immunotherapy.

A well-known epigenetic modification, DNA methylation occurs mainly in mammalian CpG islands and can regulate gene transcription to ensure cell-specific programmed gene expression (Bird, 2002; Oe et al., 2021; Singh and Edwards, 2021). Many studies have shown that abnormal DNA methylation is related to the occurrence of various diseases, including cancers (Meng et al., 2018; Zhang M. et al., 2020). The latest research has also confirmed that DNA methylation is closely related to tissue immune status (Lee et al., 2001; Thomas et al., 2012; Ghoneim et al., 2017). Delacher et al. found that an important feature of differentiated regulatory T-cell populations and lymphoid T cells in different tissues was the gain (or loss) of DNA methylation (Delacher et al., 2017). In addition, the blocking of DNA methylation has also been reported to maintain the effector functions of CD8^+^ T cells during chronic infections (Ghoneim et al., 2017). Considering this close connection between DNA methylation and immune function, we reasoned that this epigenetic modification may be involved in the ICI process against HCC. However, few studies have extensively analyzed the relationship between DNA methylation and the effects of ICIs on HCC.

Previous studies have shown that the expression of PD-1 is closely related to ICI treatment (El-Khoueiry et al., 2017; Zhu et al., 2018). There have been many studies based on the expression of PD-1 in the immune microenvironment of liver cancer to explore its role, but these studies mainly focus on samples with high PD-1 expression (Tan et al., 2019; Voutsadakis, 2019). Here, we have demonstrated the DNA-methylation characteristics of PD-1-negative HCC samples and identified 31 differentially methylated and expressed genes (DMEGs) using combined analyses of methylation, transcriptomes, and prognostic information in concert with a functional-enrichment analysis to determine the potential functions of these genes. Furthermore, we identified potential HCC therapeutic drugs based on the DrugBank database. Based on these findings, we used a LASSO (least absolute shrinkage and selection operator) regression analysis to determine a prognostic model, based on three of these DMEGs, with good predictive ability. This research provides new insights for in-depth studies of methylation in PD-1-negative HCC.

## Materials and Methods

### Data Acquisition and Processing

Data from TCGA was obtained through the TCGA Genomic Data Commons application programming interface. We obtained the most current (October 2, 2020) TCGA-LIHC expression profile data, DNA methylation data, and clinical follow-up information. Both normal samples (*n* = 50) and tumor samples (*n* = 371) were represented in TCGA data set, and 50 normal samples and 177 tumor samples were also represented in the DNA-methylation data set. HCC samples with PD-1 expression levels lower than the average found in normal samples were regarded as PD-1-negative samples. Using this criteria, 177 PD-1-negative samples were identified.

### Processing of Gene-Expression Data and DNA-Methylation Data

Gene expression data from the PD-1-negative samples was log2-converted and then analyzed for differential expression using the “limma” package in R software (Phipson et al., 2016). The *p*-values were converted to FDR values based on the Benjamini and Hochberg method. FDR > 0.01 and log2FC > 1 were considered to be up-regulated gene expression; FDR > 0.01 and log2FC < 1 were considered to be down-regulated gene expression.

The same R software package was applied to the DNA methylation data set from TCGA-LIHC to identify differentially methylated CpG genes (DMGs). Methylation intensities were represented by β values, and the threshold for DMG recognition was FDR < 0.05 and an absolute delta β-value > 0.3. We subsequently calculated average β-values for different regions, including the 5′- untranslated region (5′-UTR), first exon, gene body, 3′-UTR, TSS1500, and TSS200.

### Immune Infiltration Analysis

The “ESTIMATE” R software package was used to determine ESTIMATE scores, stromal scores, and immune scores for both the HCC and normal samples (Verhaak et al., 2010). These scores were used to describe the overall immune-cell infiltration of the microenvironment.

### Identification of DEGs and DMGs

All gene identifications based on differences in FDR < 0.01 were considered to be credible. We then performed a joint analysis of both DEGs and DMGs and divided the resulting DMEGs genes into four groups: HypoUp (β-value < -0.3 and log2FC > 1); HypoDown (β-value < -0.3 and log2FC < 1); HyperUp (β-value > -0.3 and log2FC > 1); and HyperDown (β-value > -0.3 and log2FC < 1).

### Functional Enrichment Analysis

We used the “clusterProfiler” package in R software to perform a gene ontology functional enrichment analysis and a KEGG pathway-annotation analysis of the DMGs, DEGs, and DMEGs to identify the important biological processes and pathways related to these differentially expressed genes (Yu et al., 2012).

### Screening for Potential Target Drugs

We screened the DrugBank database^1^ to identify potential drugs capable of up-regulating DMEGs. NetworkAnalyst 3.0^2^, a web-based visual analysis platform for analyzing and interpreting systems-level gene expression data, was used to analyze protein-drug interactions from the DMEGs based on the DrugBank database (Zhou et al., 2019). The HCC-drug proximity was calculated using the following formula:

d⁢(S,T)=1⁢∑t∈Tmins∈S⁢(d⁢(s,t)+ω)

where S represents the DMEGs; D represents the degree of BPH-related gene-set nodes in the PPIs; T represents the drug-target gene set; the distance d (s,t) represents the shortest path between the s node and the t node; and ω is the weight of the target gene. If the target gene was a gene in the BPH-related gene set, the calculation method was ω = -ln(D + 1), otherwise ω = 0.

### Analysis of DMEG-Related Prognostic Signature Genes

For DMEGs, we used the Principal Component Analysis method to distinguish between HCC and adjacent samples. Linear Discriminant Analysis was used to classify the samples using DMEG expression-profile data and methylation data, and the leave-one-out cross-validation method was used for verification.

To determine relationships between DMEG expressions and prognoses, we first randomly divided the PD-1-negative samples into two groups: a training set (*n* = 88) and a validation set (*n* = 89). For the DMEG-expression and clinical-survival data, we performed 1000 LASSO regression analyses, using 10-fold cross-validation, summarized the dimensionality reduction results each time, and then counted the number of times each probe appeared per 100 times.

## Results

### PD-1 Expression in HCC and Normal Tissue Samples, and Their Microenvironment Characteristics

**FIGURE 1:**
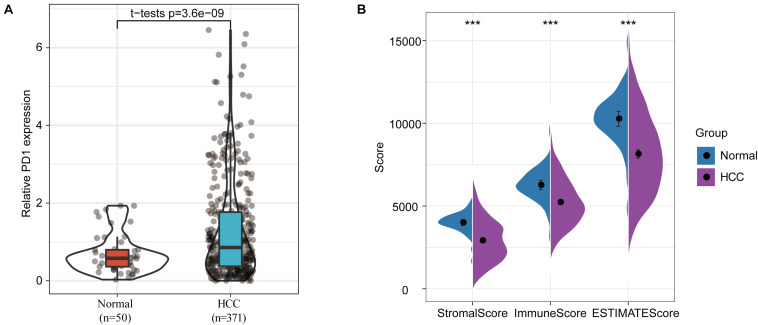
Differences in immune infiltration between normal and HCC samples. **(A)** PD-1 expression differences between normal and HCC samples. **(B)** Differences in stromal scores, immune scores, and ESTIMATE scores between normal and HCC samples, ^∗∗∗^*p* < 0.001.

A differential-expression analysis indicated that the expression of PD-1 in HCC samples was significantly higher compared to its expression in normal samples (Figure 1A). As expected, stromal and immune scores based on the ESTIMATE (Estimation of Stromal and Immune Cells in Malignant Tumors using Expression Data) analysis were both significantly reduced in HCC samples compared to normal samples (Figure 1B). These results indicate that stromal and immune infiltrations in the tumor microenvironment were significantly inhibited, which is consistent with previous studies (Binnewies et al., 2018; Ruf et al., 2021).

### Gene Analysis for Differential DNA Methylation

**FIGURE 2:**
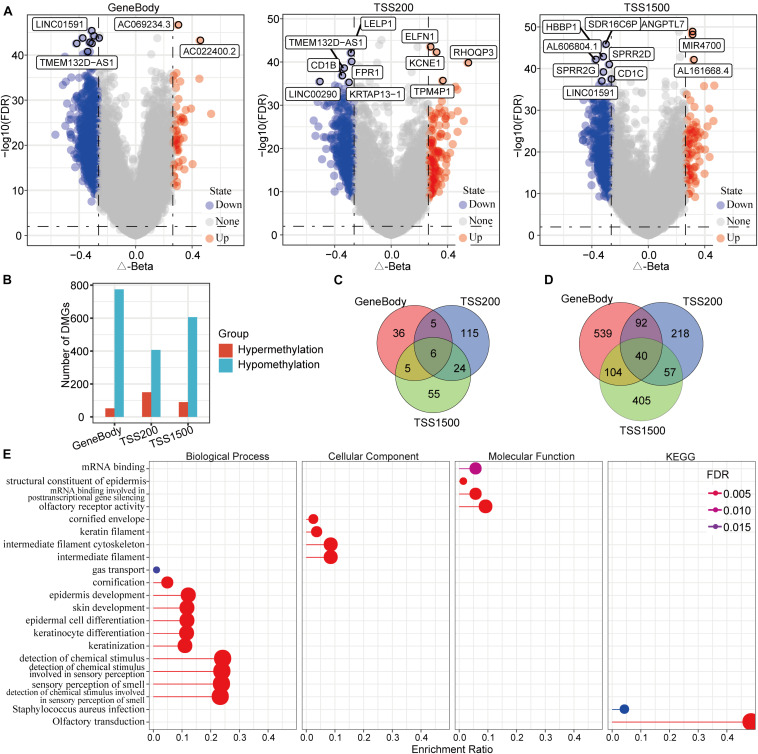
Identification and characteristics of genes with differential DNA methylation in PD-1 negative HCC samples. **(A)** A volcano map of methylation differences of the gene body, TSS200, and TSS1500 regions in PD-1 negative HCC samples. **(B)** The differentially methylated genes in these three regions were mostly hypomethylated. **(C)** A Venn diagram of hypermethylated genes in the three regions. **(D)** A Venn diagram of hypomethylated genes in the three regions. **(E)** Gene ontology functional-enrichment analysis and KEGG annotation analysis of differentially methylated genes. The most-intense red color represents the smallest FDR, and dot size is proportional to enrichment number.

To identify differentially methylated genes, we analyzed TSS200 [transcription start site (TSS) to 200 nucleotides upstream of the TSS], TSS1500 (200 to 1500 nucleotides upstream of the TSS), and gene body methylation levels in both the PD-1-negative HCC and normal samples. The results indicated a total of 1,700 differentially methylated genes in the HCC samples. Specifically, 52 hypermethylated and 775 hypomethylated genes were identified in gene body regions, 150 hypermethylated and 407 hypomethylated genes were identified in TSS200 regions, and 90 hypermethylated and 606 hypomethylated genes were identified in TSS1500 regions (Figure 2A). The number of hypomethylated genes in these regions was far greater than the number of hypermethylated genes, especially for gene body regions (Figure 2B). A set-distribution analysis of the results showed that six hypermethylated genes were represented in all three of the regions, with 206 genes represented in only one of the regions, and that 40 hypomethylated genes were represented in all three of the regions, with 1162 genes represented in only one of the regions (Figures 2C,D), suggesting that DNA methylation levels are region-specific. The functional enrichment analysis showed that these differentially methylated genes (DMGs) were mainly enriched in 11 biological processes, four cellular components, four molecular functions, and two Kyoto Encyclopedia of Genes and Genomes (KEGG) pathways (including mRNA binding, structural constituents of the epidermis, and mRNA binding involved in posttranscriptional gene silencing) (Figure 2E).

### Functional Enrichment Analysis of Differentially Expressed Genes (DEGs)

**FIGURE 3:**
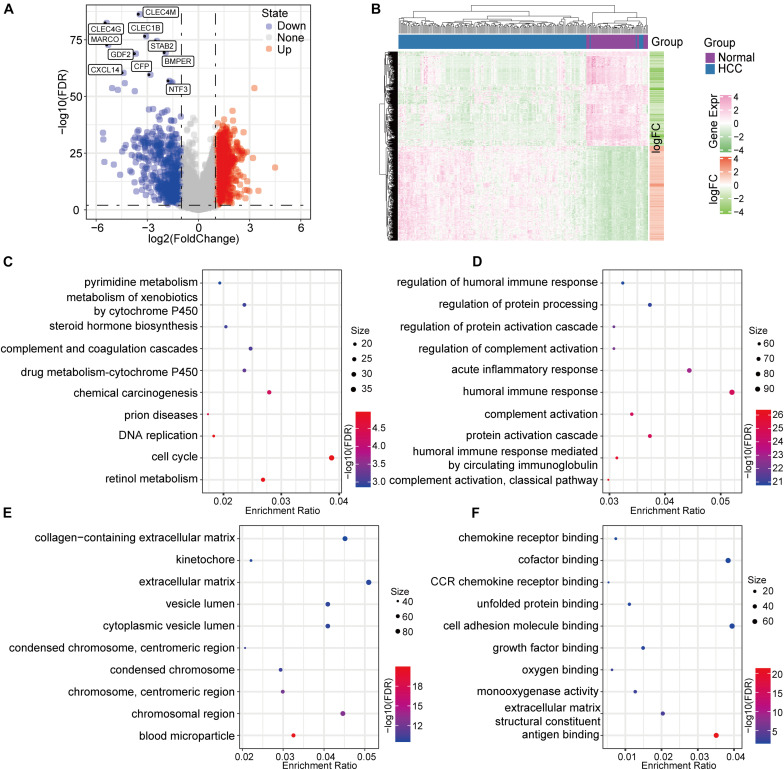
Identification and characteristics of the DEGs based on PD-1-negative HCC samples. **(A)** A volcano map of the DEGs. **(B)** A differential-expression heat map of the normal and HCC samples. **(C)** KEGG annotation analysis of the DEGs. **(D)** Gene ontology biological-function enrichment analysis of the DEGs. **(E)** DEG analysis for gene ontology cell-component enrichment. **(F)** Molecular function enrichment analysis of the DEGs.

Gene-expression profile data from 177 PD-1-negative tumor samples and 50 normal samples were used to determine differential gene expression. The screening criteria were false discovery rate (FDR) < 0.01 and log2FC (fold change) > 1. A total of 2249 differentially expressed genes (DEGs) were identified, of which 1659 were up-regulated in tumors and 590 were down-regulated (Figure 3A). A unsupervised hierarchical cluster analysis showed that these DEGs could distinguish between tumor and normal samples (Figure 3B). We performed a functional-enrichment analysis of these genes, and the results showed that a variety of tumor and immune-related pathways were enriched (e.g., cell cycle, DNA replication, complement and coagulation cascades, complement activation, and humoral immune responses) (Figures 3C–F).

### Identification of DMEGs

**FIGURE 4:**
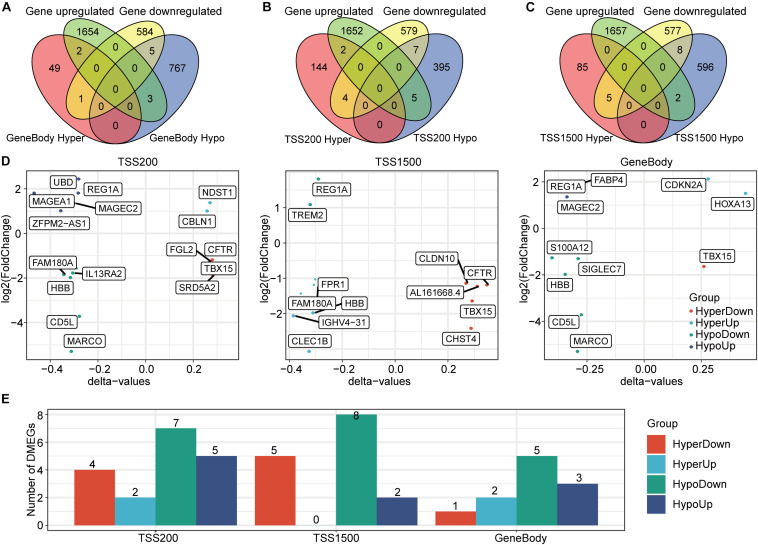
Identification and expression patterns of DMEGs. **(A)** A Venn diagram of the DEGs and the DMGs in gene body regions. **(B)** A Venn diagram of the DEGs and the DMGs in TSS200 regions. **(C)** A Venn diagram of the DEGs and the DMGs in TSS1500 regions. **(D)** Distribution of the four DMEG expression modes in the three regions. **(E)** A histogram of the four DMEG regulatory modes in the three regions.

Given the importance of both methylation and transcription in HCC occurrence and development, we jointly analyzed DMGs and DEGs to more fully explore the relationship between these two processes with the idea that genes demonstrating both differential DNA methylation and expression may play crucial HCC roles (Hu et al., 2020). A set-distribution analysis showed that 11 DMEGs were identified in gene body regions, 18 DMEGs were identified in TSS200 regions, and 15 DMEGs were identified in TSS1500 regions (Figures 4A–C). In addition, the extent to which these genes showed both differential methylation and expression is shown in Figure 4D. The observation that most of these DMEGs were hypomethylated in tumors is consistent with previous reports (Fabianowska-Majewska et al., 2021). Interestingly, several DMEGs (e.g., *TBX15*, *REG1A*, and *HBB*) were found in all three regions, indicating that these genes may have transcriptional differences caused by differential methylation. Previous reports have shown that *TBX15* expression can be used as a prognostic marker for HCC (Morine et al., 2020), and *HBB* has been reported to play a key role in prostate cancer differentiation and in a variety of important biological pathways (e.g., iron metabolism) (Chen and Sun, 2021; Lin et al., 2021). Based on the present expression data, we identified four different regulatory gene sets in the three DNA regions, for a total of 31 DMEGs (Figure 4E).

**FIGURE 5:**
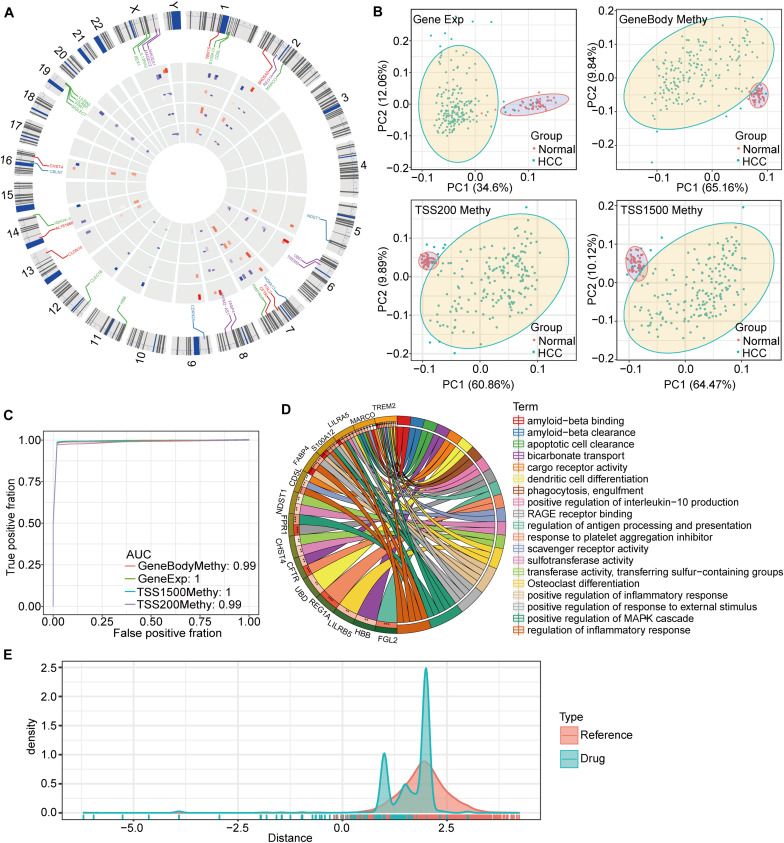
Characteristics of DMEGs and drug distance. **(A)** DMEG distributions on chromosomes. **(B)** Principal component analysis of DMEG expression and methylation in the three DNA regions. **(C)** Construction of the linear discriminant model based on the expression profiles and methylation levels of DMEGs for predicting the ROC curves for HCC and normal samples. **(D)** The results of KEGG pathway-annotation and gene ontology enrichment analyses for the DMEGs, with different colors representing different pathways, and the lines indicating relationships between genes and pathways. **(E)** A drug distance-density plot for the differentially methylated and expressed gene set.

To better understand the potential functions of these genes in HCC, an in-depth chromosome (chr) distribution analysis was carried out (Figure 5A). We found that chr7, chr18, and chrX had the most DMEGs. Interestingly, the methylation patterns of the DMEGs in adjacent chromosomal gene regions were roughly consistent, suggesting that these genes may have cooperative expression patterns and functions. In order to verify gene effectiveness, we used the expression profiles of these 31 DMEGs and the methylation data from the DMEGs in each of the three regions to construct a linear judgment classification model. The results showed that these DMEGs could effectively distinguish PD-1-negative HCC samples from normal samples (Figure 5B). The corresponding receiver operating characteristic (ROC) curve analysis showed an area-under-the-curve (AUC) value ≥0.99 (Figure 5C). The functional enrichment analysis showed that DMEGs were mainly enriched in amyloid and apoptotic-cell clearance, as well as in the activation of signaling pathways, such as mitogen-activated protein kinase pathways (Figure 5D).

### Identification of Potential Target Drugs Based on PD-1-Negative DMEGs

A DMEG protein-drug interaction analysis was performed using the NetworkAnalyst 3.0 tool based on the DrugBank database, and 6 genes were found to interact with drugs (Table 1). Among them, cystic fibrosis transmembrane conductance regulator (CFTR) and hemoglobin subunit beta (HBB) proteins had the most drug interactions: 12 with HBB (e.g., pentaerythritol tetranitrate, and 4-carboxycinnamic acid) and 7 with CFTR (e.g., colforsin, crofelemer, and lonidamine). Some of these identified drugs may be effective against HCC.

**TABLE 1:** The interaction between DMEGs and drugs.

**Gene**	**Gene type**	**Drug count**	**Drug example**
CFTR	HyperDown	7	Colforsin; Crofelemer; Lonidamine
HBB	HypoDown	12	4-Carboxycinnamic Acid; Pentaerythritol tetranitrate; 2-[(2-methoxy-5-methylphenoxy) methyl] pyridine
IL13RA2	HypoDown	1	AER001
MARCO	HypoDown	2	Titanium dioxide; Silicon dioxide
S100A12	HypoDown	1	Amlexanox
SRD5A2	HyperDown	3	Azelaic acid; Dutasteride; Finasteride

Based on these drug-target pairs from DrugBank, as well as the string key protein-protein interaction (PPI) network (threshold score was set at 600), we calculated drug-HCC proximities. For both our DMEGs as samples, and for randomly selected genes as samples, we found that the number of drugs was significantly reduced when drug distance was less than 0.8 (Figure 5E). This suggests that when HCC-drug proximity is less than 0.8, the drug may have a targeted impact on the disease.

### Molecular Docking Analysis Verifies the Affinity of Candidate Drugs

**FIGURE 6:**
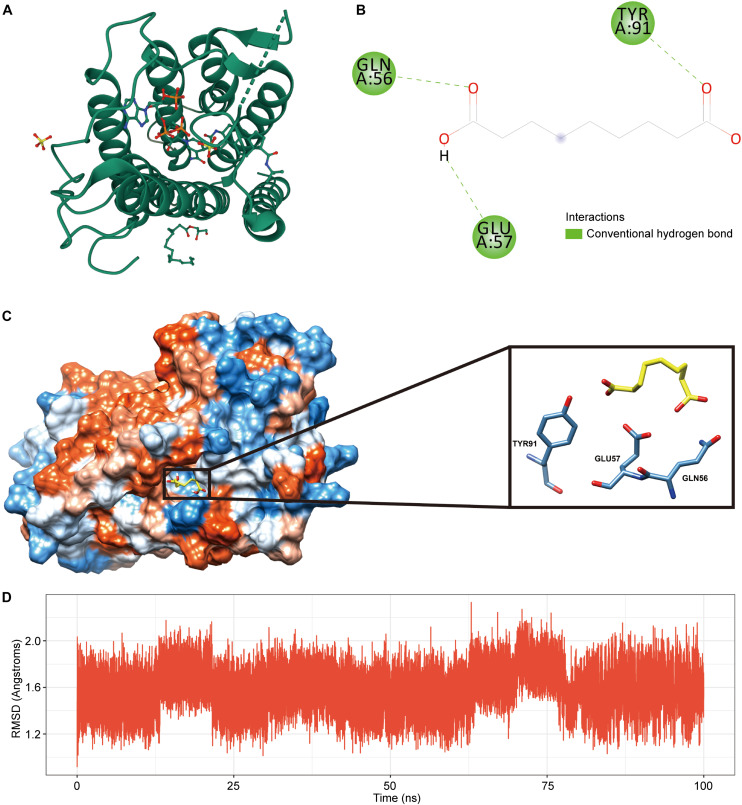
SRD5A2- Azelaic acid molecular docking analysis. **(A)** 3D model of SRD5A2 protein structure. **(B)** 2D interaction diagram of Azelaic acid and SRD5A2 protein. **(C)** The binding domain of Azelaic acid and SRD5A2 protein, in which hydrogen bonds are shown in green and hydrophobic interactions are shown in pink. Amino acid residues are displayed as steel blue. **(D)** The curve of the RMSD value of the protein backbone during molecular dynamics in 100ns.

Considering the accuracy of molecular docking, we chose SRD5A2 with a moderate molecular weight as a representative to perform molecular docking analysis in order to clarify the binding model between drug candidates and gene targets. We first downloaded the 3D model of SRD5A2 protein (PDB ID: 7BW1) from the PDB database for molecular docking experiments (Figure 6A). Autodock Vina molecular docking results show that the compound can bind tightly to the active site of the SRD5A2 protein, with a molecular docking score of -6.5kcal/mol (Figure 6B). In addition, we found that the compound could generate favorable hydrogen bonds with the important amino acid residues GLU57, GLN56 and TYR91 in the SRD5A2 protein, as shown in Figure 6C. The above results suggest that the drug Azelaic acid can interact closely with the SRD5A2 protein, thereby affecting the activity of the SRD5A2 protein. Meanwhile, we used molecular dynamics simulation to further evaluate the stability of the protein model combined with the drug, and used the RMSD method to estimate the stability of the protein model (Figure 6D). During the 100ns molecular dynamics simulation, we can find that the protein-backbone is maintained in a relatively stable state as a whole, indicating that the protein is relatively stable during the molecular dynamics simulation.

### Prognostic Genetic Signature of DMEGs in PD-1-Negative Samples

**FIGURE 7:**
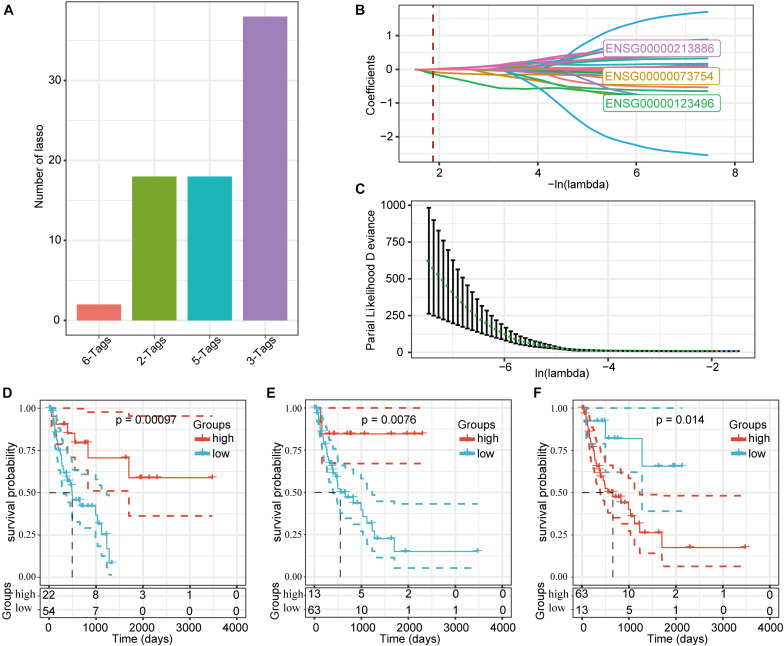
Prognostic gene signatures related to DMEGs. **(A)** LASSO-regression frequencies for each combination of genes. **(B)** Change-coefficient trajectories for each of the three probe genes using different lambdas. **(C)** The distribution of standard deviations in this model under different lambda conditions. **(D)** Kaplan-Meier curves for the *CD5L* high- and low-expression groups. **(E)** Kaplan-Meier curves for the *CD213A2* high- and low-expression groups. **(F)** Kaplan-Meier curves for the *UBD* high- and low-expression groups.

We used a LASSO regression analysis to reduce the dimensionality of the expression and prognostic data for these DMEGs, and obtained a combined maximum frequency for three probe genes (Figure 7A; ENSG00000073754, ENSG00000123496, and ENSG00000213886). The trajectories for these three genes with different lambdas are shown in Figure 7B, and the standard-deviation distribution of the different lambdas is shown in Figure 7C. The survival-model results demonstrated that, with a median cutoff, the high-expression group was significantly different from the low-expression group using these three genes, indicating a highly effective model (Figures 7D–F). According to the LASSO analysis, the determination formula was:

RiskScore=-0.253×ENSG00000073754+0.111×ENSG00000213886-0.843×ENSG00000123496

Detailed information about these three genes is presented in Table 2. Both *CD5L* and *CD213A2* were determined to be HCC protective factors, and *UBD* was determined to be a risk factor.

**TABLE 2:** LASSO identifies 3 prognostic-related DMEGs.

**ENSG id**	**Gene symbol**	**P value**	**HR**	**Low 95% CI**	**High 95% CI**
ENSG00000073754	CD5L	0.005001056	0.7130075	0.5630112	0.9029655
ENSG00000123496	CD213A2	0.019657250	0.3175769	0.1211487	0.8324900
ENSG00000213886	UBD	0.021742632	1.2143576	1.0287460	1.4334583

**FIGURE 8:**
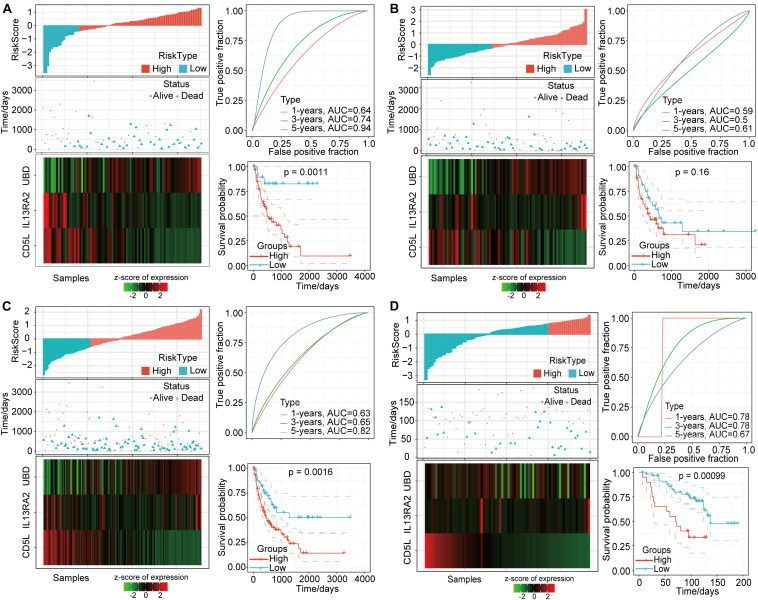
Characteristics of prognostic gene signatures based on 3 DMEGs. **(A)** Training-set relationships between risk scores, survival status, and gene expressions for the three DMEGs and prognostic-signature ROC analysis and Kaplan-Meier curves. **(B)** Validation-set relationships between risk scores, survival status, and gene expressions for the three DMEGs and prognostic-signature ROC analysis and Kaplan-Meier curves. **(C)** The total TCGA-LIHC-set PD-1 negative samples relationships between risk scores, survival status, and gene expressions of the three DMEGs and prognostic-signature ROC analysis and Kaplan-Meier curves. **(D)** The GSE10141-set PD-1 negative samples relationships between risk scores, survival status, and gene expressions of the three DMEGs and prognostic-signature ROC analysis and Kaplan-Meier curves.

According to the above formula, we calculated the risk score for each sample and determined corresponding survival status and expression changes for the three different signature genes as risk values increased (Figure 8A). We found that most of the training-set samples had higher risk scores, and that samples with high-risk scores had worse prognoses. In addition, *UBD* expression was found to increase with increasing risk-score values, while *CD5L* and *CD213A2* expressions decreased with increasing risk scores. Furthermore, we conducted ROC analyses for the prognostic classification of risk scores, and the prognostic prediction classification efficiencies for 1 year, 3 years, and 5 years were 0.64, 0.74, and 0.94, respectively (Figure 8A). These data indicate that this model was highly predictive for long-term survival. In addition, based on risk-score value z-scores, we divided the samples into high- and low-risk groups. The survival curves showed very significant differences between these groups (log rank *P* = 0.0011), in which 57 of the samples were classified as high-risk and 19 samples were classified as low-risk. We also conducted an analysis of the validation set using the same model and coefficients as for the training-set analysis. The validation results showed that the expression trends of these three signature genes were consistent with the training set (Figure 8B). Compared to the low-risk group, the overall survival rate in the high-risk group was worse, but this difference was not statistically significant.

In order to further verify the effectiveness of this gene-signature model, we applied it to all PD-1 low-expression samples in the Cancer Genome Atlas (TCGA) and GSE10141 HCC data set. Similar to the previous results, most of these samples had higher risk scores that related to lower survival status (Figure 8C). The ROC analysis showed that the AUC values at 1, 3, and 5 years were 0.63, 0.65, and 0.82, respectively (Figure 8C). At the same time, the overall survival rates for the high-risk group and the low-risk group were also significantly different (*P* = 0.0016, Figure 8C). The GSE10141 data set results showed that more samples were identified as having lower-risk scores, and that the 1-year, 3-year, and 5-year AUC values were 0.78, 0.78, and 0.67, respectively (Figure 8D). In addition, the prognoses for the high-risk group and the low-risk group were significantly different (*P* = 0.00099). These differences in prognostic scores and expression levels may have been due to the batch effect on the different platforms.

## Discussion

With the development of ICIs, immunotherapy has become the new focus of attention in the field of tumor treatments (Bersanelli et al., 2021). However, only a small proportion of HCC patients actually respond to ICIs, and one of the important reasons for this may be HCC expression of PD-1 and the infiltration of CD4^+^ and CD8^+^ T cells (Sia et al., 2017; Kurebayashi et al., 2018; Kudo, 2020a). Previous studies have shown that DNA methylation can affect the immune status of the tumor microenvironment and tumor responses to ICIs, and that a lack of DNA methylation is related to immune-evasion characteristics (Duruisseaux et al., 2018; Jung et al., 2019). Therefore, it is of instructive significance to investigate the changes in DNA methylation in PD1-negative HCC samples on the loss of the anti-tumor effect of ICIs and to further search for other therapeutic targets.

Here, using the Liver Hepatocellular Carcinoma (LIHC) cohort from TGCA, we found that stromal-score, immune-score, and ESTIMATE-score values for tumor samples were significantly lower compared to normal samples, and that PD-1 expression was higher compared to normal samples. These differences highlight the immunosuppressive state in these tumors. Using PD-1-negative samples for screening, we identified DEGs and DMGs in three DNA regions, and a functional enrichment analysis showed that they were related to tumor immunity and cancer-related cell pathways (Hu et al., 2020). As there is evidence that DMEGs play a key role in tumorigenesis, we conducted a joint DEG-DMG analysis and identified 31 gene candidates. We then divided these DMEGs into four groups based on modes of expression. Interestingly, most DMEGs demonstrated hypomethylation. Consistent with these results, studies have shown that overall demethylation and hypomethylation of both oncogenes and metastasis-promoting genes are characteristics common to almost all cancers, including HCC (Zhao et al., 2020; Fabianowska-Majewska et al., 2021). The chromosomal distribution of these genes showed that DMEGs in adjacent gene regions had similar expression patterns, suggesting that adjacent regions may be regulated by the same methylases/demethylases. In addition, these 31 DMEGs could distinguish between tumor and normal samples based on methylation and expression levels, indicating their potential importance.

The protein-drug interaction analysis provided another perspective for evaluating the potential therapeutic effects of DMEGs on HCC. Conductance regulator and *HBB* were identified as the DMEGs having the most drug interactions. Conductance regulator encodes chloride ion and bicarbonate ion channels and has been implicated in a variety of cancers. It has also been identified as a molecular biomarker for early HCC diagnosis (Hogan, 1999; Moribe et al., 2009). Hemoglobin subunit beta has also been reported to be a diagnostic biomarker in cancers (Shi et al., 2018). Here, we found that these genes interacted with a variety of drugs, including the commonly used anti-tumor drug Lonidamine, a mitochondrial hexokinase inhibitor, which can inhibit the glycolysis of tumor cells. The drugs identified using the analysis above may provide alternative ways to treat HCC. In addition, we found that when the drug-DMEG distance was less than 0.8, drug interactions were significantly reduced, suggesting that 0.8 may represent an important threshold. With distances <0.8, the corresponding drugs may have more precise targeting effects for HCC treatment.

In order to evaluate the predictive power of these DMEGs for HCC prognosis, we used a LASSO regression to determine a prognostic model for HCC based on three genes (*UBD*, *CD5L*, and *CD213A2*) that can modulate immune responses. Ubiquitin D (UBD), a ubiquitin-like protein modifier, binds to target proteins by covalent bonding and then causes them to be degraded by the 26s proteasome (Guarascio et al., 2020). Studies have shown that UBD can regulate the activation of the tumor necrosis factor alpha (TNF-α) -induced, and lipopolysaccharide-mediated, innate immune response central mediator nuclear factor kappa B (NF-κB), by promoting TNF-α-mediated ubiquitinated-I-κB-α proteasome degradation (Kawamoto et al., 2019). We speculate that increased *UBD* expression may promote an immune response in the tumor microenvironment, which suppresses tumor growth. The cysteine-rich inflammatory regulator CD5L has been shown to promote proliferation and activate autophagy in HCC by binding heat shock-A5 proteins (Armengol et al., 2013; Sanjurjo et al., 2015; Aran et al., 2018). Consistent with previous studies, the present results show that *CD5L* expression is a risk factor for HCC and may help researchers to reinterpret its role from the new perspective of methylation and immunity. CD213A2 has been shown to bind to interleukin-13 (IL-13) and activate its immunomodulatory function (Tabata and Khurana Hershey, 2007); however, no direct tumor-related role has been found, so further study of *CD213A2* and its effects on HCC, including through methylation and immunity, is warranted.

The tumor’s response to ICIs largely depends on the state of the tumor microenvironment. As mentioned above, the lack of DNA methylation is related to the immune evasion characteristics of the tumor microenvironment. Here, we explored the DNA methylation characteristics and potential functional pathways of PD1-negative HCC patients, and identified the genes that play a key role in this process. These genes participate in the tumor immune microenvironment through possible DNA methylation regulation and further affect the anti-tumor effect of ICIs. We have proposed a model for determining PD-1-negative HCC prognoses based on these three genes. This model was clearly able to divide PD-1-negative HCC samples into high- and low-risk groups, with clear trends for DMEG expressions, and significantly different prognoses between these two groups. The use of an independent verification queue also confirmed its effectiveness. Therefore, further exploring the role of these hub genes in this process will help guide researchers to have a deeper understanding of the PD1-negative tumor microenvironment. These hub genes are also expected to become potential targets for enhancing the efficacy of ICIs.

## Conclusion

The present research has revealed the methylation/transcription characteristics of PD-1-negative HCC samples and identified potential therapeutic targets and drugs. Most importantly, we have demonstrated the effectiveness of a prognostic model for HCC based on three DMEGs. These results provide insights into potential treatment strategies for HCC that are not sensitive to PD-1 inhibitors and into mechanisms by which methylation may affect HCC.

## Data Availability Statement

Publicly available datasets were analyzed in this study. The names of the repository/repositories and accession number(s) can be found in the article/supplementary material.

## Author Contributions

WG designed the study, wrote this manuscript, and collected samples. LZ searched the articles and made figures. Both authors read and approved the final manuscript.

## Conflict of Interest

The authors declare that the research was conducted in the absence of any commercial or financial relationships that could be construed as a potential conflict of interest.

## Publisher’s Note

All claims expressed in this article are solely those of the authors and do not necessarily represent those of their affiliated organizations, or those of the publisher, the editors and the reviewers. Any product that may be evaluated in this article, or claim that may be made by its manufacturer, is not guaranteed or endorsed by the publisher.

## Footnotes

1
https://www.drugbank.com/

2
http://www.networkanalyst.ca/

## Abbreviations

HCC: hepatocellular carcinoma
ICIs: immune checkpoint inhibitors
PD-1: programmed cell death protein 1
LASSO: least absolute shrinkage and selection operator
DMEGs: differentially methylated and expressed genes
ESTIMATE: Estimation of Stromal and Immune Cells in Malignant Tumors using Expression Data
DMGs: differentially methylated genes
KEGG: Kyoto Encyclopedia of Genes and Genomes
DEGs: differentially expressed genes
TCGA: Cancer Genome Atlas
LIHC: liver hepatocellular carcinoma
UBD: Ubiquitin D
TNF-α: tumor necrosis factor alpha
NF-κB: nuclear factor kappa B
IL-13: interleukin-13.
